# Computational analysis of hub genes associated with sarcopenia: integrative transcriptome insights from an Asian cohort

**DOI:** 10.17179/excli2025-9087

**Published:** 2026-01-02

**Authors:** Jae Gyu Kim, Ashish Ranjan Sharma, Yeon-Hee Lee, Min-Jee Kwon, Chiranjib Chakraborty, Jin-Chul Kim, Holger Jahr, Sang-Soo Lee

**Affiliations:** 1 Institute for Skeletal Aging & Orthopedic Surgery, Hallym University-Chuncheon Sacred Heart Hospital, Chuncheon-si, 24252, Gangwon-do, Republic of Korea; 2 Department of Biotechnology, School of Life Science and Biotechnology, Adamas University, Barasat-Barrackpore Road, Kolkata, 700126 West Bengal, India; 3 Department of Biomedical Science & Institute of Bioscience and Biotechnology, Kangwon National University, Chuncheon-si, 24341 Gangwon-do, Republic of Korea; 4 Institute of Structural Mechanics and Lightweight Design, RWTH Aachen University, 52062 Aachen, Germany; 5 Winsløw Unit of Anatomy, Histology and Plastination, Department of Molecular Medicine, University of Southern Denmark, 5000 Odense, Denmark

**Keywords:** osteosarcopenia, biomarkers, transcriptomics, autophagy, inflammation

## Abstract

Sarcopenia, a progressive loss of skeletal muscle mass and strength, leads to frailty, falls, fractures, and delayed recovery following orthopedic surgery. When combined with osteoporosis, it manifests as osteosarcopenia, exacerbating musculoskeletal fragility. Although chronic inflammation, mitochondrial dysfunction, and impaired autophagy are recognized contributors, the integrated regulation of these processes in Asian populations remains unclear. This study aimed to elucidate molecular mediators and signaling pathways connecting inflammation, autophagy, and muscle-bone degeneration using an integrated clinical-transcriptomic approach. Transcriptomic data (GSE226151) comprising vastus lateralis muscle samples from 20 sarcopenic patients and 20 age- and sex-matched healthy Asian controls were analyzed using ExDEGA, with differentially expressed genes (DEGs) defined by |log₂ fold change| ≥ 1 and FDR < 0.05. Functional enrichment via ShinyGO identified key Gene Ontology and KEGG pathways, while STRING-Cytoscape network analysis revealed four hub genes-ADAM8, BECN1, KLF4, and GBP5-with high connectivity (degree >10) enriched in cytokine-cytokine receptor interaction and PI3K-Akt pathways. Gene Set Enrichment Analysis further validated these associations. The expression of these hub genes inversely correlated with skeletal muscle index (r = -0.63 to -0.74; p < 0.01) and grip strength (r = -0.58 to -0.69; p < 0.05). Clinically, sarcopenic individuals exhibited significantly lower BMI, gait speed, and muscle mass (all p < 0.001). Integrating bioinformatics and clinical data identified these four genes as critical mediators linking inflammation, defective autophagy, and musculoskeletal decline in sarcopenia. These findings provide translational insight into the molecular mechanisms underlying osteosarcopenia and suggest potential biomarkers and therapeutic targets to improve diagnosis and treatment in aging-related musculoskeletal disorders.

See also the graphical abstract(Fig. 1).

## Introduction

Sarcopenia is a progressive skeletal muscle disorder characterized by severe outcomes, such as falls, fractures, physical disabilities, and increased mortality, as defined by the European Working Group on Sarcopenia in Older People 2 (EWGSOP2) (Cruz-Jentoft et al., 2019[4]). A complex interaction between genetic and environmental factors, such as lifestyle, physical activity, dietary practices, depletion of satellite cells (myogenic stem cells), changes in muscle protein homeostasis, anabolic resistance, and neuromuscular dysfunction, results in this multifactorial condition. Due to its impact on age-related fragility and quality of life, sarcopenia has become an important musculoskeletal condition in the modern super-aging era (Papadopoulou, 2020[18]). Patients with hip fractures and sarcopenia had a higher mortality rate than those without sarcopenia (Kim et al., 2022[11], Portnoy et al., 2024[21]). The 1-year, 2-year, and 5-year mortality rates were all significantly higher in the sarcopenia group. Studies have highlighted that osteosarcopenia patients (a combination of osteoporosis and sarcopenia) demonstrate a 1.8-fold higher mortality rate than non-osteosarcopenia patients at the 1-year follow-up (Alexiou et al., 2024[2]). Additionally, a paradigm shift toward an integrated understanding of these mechanistically linked diseases is reflected in the recent surge in research interest in osteosarcopenia, a pathophysiological condition characterized by concomitant deterioration of bone (osteopenia/osteoporosis) and skeletal muscle (Sharma et al., 2024[24]). 

The etiology of sarcopenia is multifactorial, involving age-related hormonal changes, reduced physical activity, inadequate nutrition, and persistent low-grade inflammation (Sharma et al., 2024[24]). An increase in the secretion of inflammatory molecules within muscle cells leads to increased protein degradation, ultimately contributing to muscle atrophy. The activation of catabolic pathways, such as NF-κB and FoxO, by chronic inflammation, often known as "inflammaging," promotes protein breakdown and hinders muscle regeneration (Antuna et al., 2022[3]). Sarcopenia develops at the cellular and molecular levels due to a combination of mechanisms, including genomic instability, mitochondrial dysfunction, disrupted proteostasis, and stem cell depletion. Mitochondrial dysfunction is a key metabolic characteristic of sarcopenia, characterized by reduced oxidative phosphorylation, ATP depletion, and increased reactive oxygen species production (Kim et al., 2023[12]). Satellite cells, the intrinsic muscle stem cells, exhibit senescence-like traits in aged muscle, thereby reducing regenerative capacity. Cellular senescence negatively affects the function and number of satellite cells, thus significantly contributing to the development of sarcopenia. Additionally, impaired energy metabolism is essential for the pathogenesis of sarcopenia (Daily et al., 2022[5]). Autophagy, a quality-control mechanism regulated by Beclin-1 and the PI3K complex, is dysregulated with aging, leading to the accumulation of damaged mitochondria and compromised cellular recycling (Kang et al., 2011[10]). 

Recent evidence highlights the connection between skeletal muscle and bone in the context of aging, leading to the concept of osteosarcopenia, characterized by the concurrent decline of both tissues via common molecular and endocrine mechanisms. The interaction between muscle-derived myokines and bone-derived factors plays a significant role in systemic metabolic regulation, highlighting the intricacies of musculoskeletal aging (Sharma et al., 2024[24]). Despite these insights, substantial gaps in understanding population-specific processes of sarcopenia persist. Numerous omics-based investigations have primarily focused on Western cohorts, yet Asian populations remain underrepresented, despite their distinct genetic, nutritional, and environmental characteristics (Yuan et al., 2023[30]). Differences in muscle biology across ethnic groups may influence disease risk and biomarker expression. Traditional diagnostic techniques, such as muscle biopsies and imaging, are invasive and unsuitable for early or extensive screening (Wilson et al., 2018[27]).

Advanced approaches, particularly bioinformatics techniques, have emerged as powerful tools for elucidating the molecular mechanisms underlying complex diseases, enabling the discovery of potential disease-specific biomarkers for improved diagnostics and targeted therapies (Huang et al., 2024[8]). Integrated bioinformatics analyses, including differentially expressed gene (DEG) analysis, Gene Ontology (GO), and Kyoto Encyclopedia of Genes and Genomes (KEGG) pathway enrichment analysis, are budding techniques and can be utilized for analyzing the complex mechanisms of sarcopenia. For instance, transcriptomic analysis of muscle biopsy samples from sarcopenia patients has identified key candidate genes and dysregulated pathways, providing critical insights into sarcopenia pathogenesis (Jiao et al., 2023[9]). However, most large-scale bioinformatics studies have primarily focused on Western cohorts, often neglecting genetic, epigenetic, and environmental factors specific to non-Western cultures. The lack of ethnic diversity in omics databases reduces the relevance of results, such as biomarker discovery or therapeutic targets, to Asian and other underrepresented communities, therefore increasing discrepancies in precision medicine (Petermann-Rocha et al., 2020[20]).

A few of the Asian studies have been conducted, and have tried to explore transcriptomic or multi-omics signatures in Asian populations, but have lacked in a few areas, such as transcriptomics, to link SNPs to gene networks, have not conducted a wide profiling of differentially expressed genes, and have had a small sample size (n=20) with no multi-cohort integration (Minamino et al., 2021[17], Wu et al., 2021[28]). Thus, this study was undertaken to analyze transcriptomic data from the Genomic Spatial Event database, comprising 40 samples from patients with sarcopenia and healthy Asian controls, and to conduct comprehensive bioinformatic analyses. Moreover, research aims to improve the molecular understanding of sarcopenia by focusing on population-specific transcriptomic signatures, thereby laying the groundwork for the development of early diagnostic biomarkers and targeted therapeutic strategies for age-related muscle deterioration. 

## Methods

### Data Collection and Processing for Sarcopenia Biomarker Identification

The Gene Expression Omnibus (GEO) is a comprehensive open-access database that indexes microarray and high-throughput gene expression data submitted by research institutions worldwide (Patra et al., 2020[19]). This study utilized data from a GEO series focused on sarcopenia. The GSE226151 series was retrieved from the GEO database (GEO - NCBI, nih.gov) on February 22, 2024 (Li et al., 2023[14]). Participants were recruited from West China Hospital of Sichuan University, including 20 individuals with Sarcopenia (S) and 20 healthy aged (HA) controls matched for age and sex. Sarcopenia was diagnosed based on the clinical criteria established by the Asian Working Group on Sarcopenia in Older People 2019 (AWGSOP 2019), which includes a low skeletal muscle index (SMI) measured via bioelectrical impedance analysis (BIA), reduced grip strength, and slowed gait speed (Li et al., 2023[14]) (Table 1(Tab. 1), and supplementary Table S1). Sarcopenia was diagnosed when both the muscle strength and mass were below the defined thresholds.

Vastus lateralis muscle samples were collected from all participants, and multi-omics analyses were conducted to investigate the molecular basis of sarcopenia. The dataset was re-analyzed to identify potential biomarkers associated with sarcopenia.

### DEG analysis

To identify the DEGs involved in sarcopenia, the GSE226151 dataset was processed and converted into a differential expression analysis (ExDEGA)-compatible format using Python. ExDEGA software, developed and distributed by eBiogen, Inc., was used to generate a data matrix integrating metadata and raw count values from the S and HA groups. The HA group was designated as the reference condition to identify the DEGs in the S group. The Benjamini-Hochberg method was employed to ascertain statistical significance while controlling for the false discovery rate (FDR). Genes were classified as differentially expressed if they satisfied the criteria of |log₂ fold change| ≥ 1 and an adjusted p-value (FDR) < 0.05. Volcano and scatter plots were generated with ExDEGA to illustrate upregulated and downregulated genes, with all expression values presented as mean-normalized fold changes.

### KEGG and GO analysis (using ShinyGO 0.8 server)

Functional enrichment analysis was conducted to explore the biological functions and pathways associated with DEGs. KEGG pathway analysis was performed using the ShinyGO 0.8 server. The genes identified in the DEG analysis were used as inputs for the server to map the relevant pathways. GO analysis was performed to classify DEGs into three main categories: biological processes, cellular components, and molecular functions. This provides detailed insights into the roles, interactions, and hierarchical organization of the identified genes. Pathway information was obtained from the KEGG database. Enrichment results were filtered according to FDR-adjusted p < 0.05. Pathway visualization utilized tree maps and network diagrams, with node size reflecting enrichment magnitude (-log10 p-value).

### Search tool for the retrieval of interacting genes/proteins (STRING) analysis

Forty candidate DEGs were submitted to the STRING database (https://string-db.org/) for the prediction of protein-protein interactions (PPIs). The threshold for the confidence score was established at ≥ 0.7. The interaction networks were imported into Cytoscape (version 3.10.2) and analyzed with the ClueGO and CytoHubba plug-ins to identify key nodes and functional clusters. Hub genes are identified as nodes that rank in the top 5 % for degree or betweenness centrality (≥10 direct connections), indicating their status as the most interconnected and biologically significant proteins within the network. The multi-protein analysis option was used to enhance visualization and ensure a suitable number of interactions were presented without excessive clutter. In the resulting PPI network, the central protein is highlighted in red, whereas strongly interacting proteins are represented with bold lines.

### Statistical and bioinformatic integration

A multi-level statistical workflow was implemented to ensure the robustness of hub gene identification. Outputs from ExDEGA, functional enrichment assessment (ShinyGO), and network topology evaluation (STRING-Cytoscape) were cross-compared. Genes were classified as hub genes based on significant expression (|log₂FC| ≥ 1, FDR < 0.05), high network connectivity (degree ≥ 10), and recurrent pathway enrichment in Gene set enrichment analysis (GSEA) (NES ≥ 1, FDR < 0.25). Four genes-ADAM8, BECN1, KLF4, and GBP5-met the specified multi-criteria conditions and were identified as the primary regulatory candidates linked to sarcopenia.

### Gene Set Enrichment Analysis (using a dedicated program)

For GSEA, a Gene Classification File (GCF) was created in Excel, containing all genes from the dataset, with LOG2 values excluded and only the unique FOLD values retained. A phenotype file (CLS file) was generated to categorize the gene expression data for analysis. These files were uploaded to the GSEA platform for enrichment analysis, which enabled the identification of significant biological functions associated with the dataset. GSEA was performed with 10,000 permutations to guarantee analytical rigor. Gene sets were deemed significantly enriched when the normalized enrichment score (NES) was>1, with a nominal p-value < 0.05 and a false discovery rate (FDR) < 25 %. The leading gene sets were ranked according to NES and visualized for key pathways, including cytokine-cytokine receptor interaction, PI3K-Akt signaling, and autophagy regulation.

### PPI network construction

PPI networks were constructed to visualize the relationships between proteins. Common DEGs, key genes from weighted gene co-expression network analysis (WGCNA), and candidate genes were uploaded to STRING (https://string-db.org/) to build the PPI network with a confidence score threshold set at ≥ 0.7. Pathway analysis of candidate genes was subsequently conducted in Cytoscape (version 3.10.2), a widely used network visualization tool. STRING (version 12.0) is a comprehensive PPI database that includes over 14,000 species, 67 million proteins, and 20 billion interactions, making it an invaluable resource for functional discoveries. The minimum interaction confidence score was set to 0.7 to ensure inclusion of only high-confidence protein interactions. 

### Identification and analysis of hub genes

Hub genes are critical components within a PPI network and occupy central positions owing to their high degree of connectivity, as indicated by their nodes, edges, and interconnections. ClueGO (version 2.5.10), an advanced plugin for Cytoscape, was used to identify these hub genes. ClueGO is designed to efficiently extract and analyze the core elements of biological networks (Yang et al., 2024[29]), enabling the identification of functional clusters and pathways associated with sarcopenia. GeneMANIA (Franz et al., 2018[7]), a versatile and user-friendly tool for gene function analysis, was used to construct co-expression networks for the identified hub genes.

## Results

### Identification of DEG

**Figure 2:**
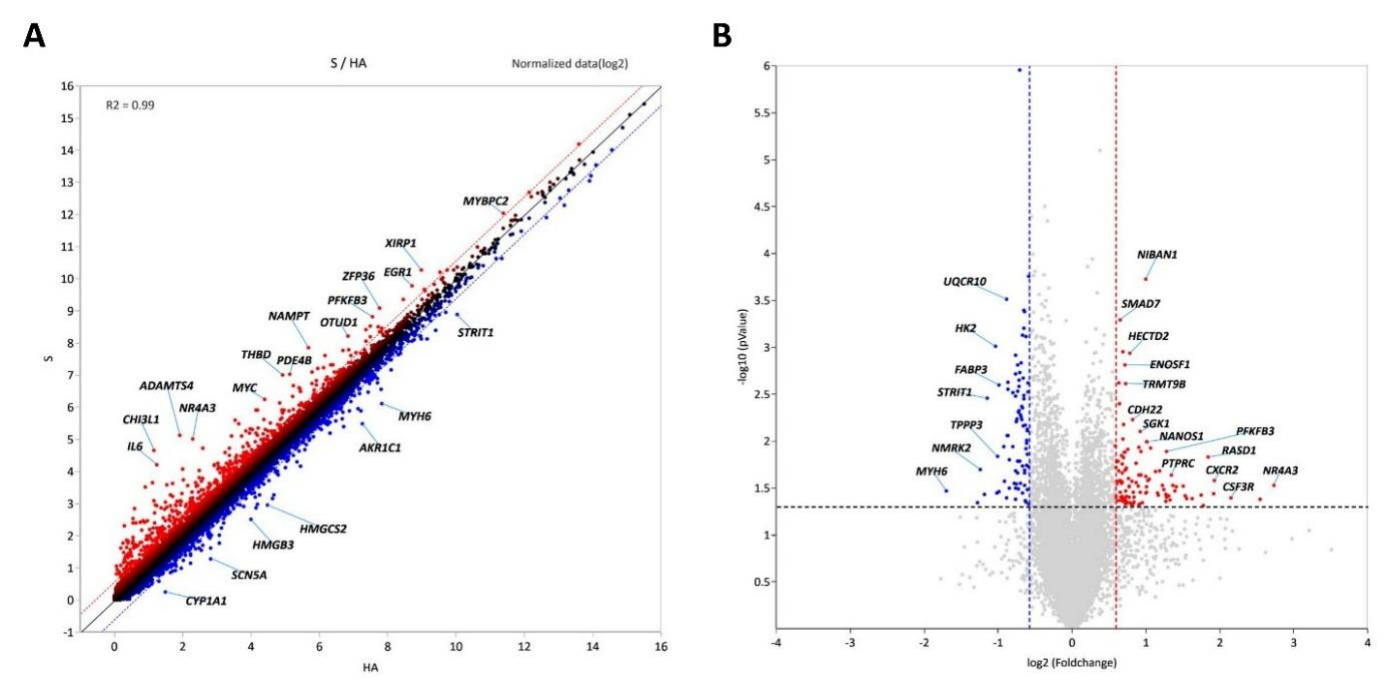
Differentially expressed genes (DEG) identified in the GSE226151 dataset. (A) Scatter plot showing DEGs identified in samples from patients with Sarcopenia (S; n = 20) compared with healthy aged control samples (HA; n = 20). Upregulated genes in sarcopenia are shown in red and downregulated genes in blue. A total of 20 upregulated and seven downregulated genes were identified relative to the reference line. (B) Volcano plot of DEGs identified between the S and HA groups. The upregulated genes are shown in red, and the downregulated genes are indicated in blue.

A comparative analysis was performed between sarcopenic (S) and healthy aged (HA) groups to identify DEGs. Scatter and volcano plots (Figure 2A-B(Fig. 2)) demonstrated distinct expression profiles among the groups. Forty genes fulfilled the significance criteria (|log₂FC| ≥ 1, adjusted p < 0.05). Several genes, including MYBPC2, XIRP1, EGF1, PFKB3, nd NAMPT, exhibited marked upregulation, while MYH6, AKR1C1, and HMGCS2 showed significant downregulation. The volcano plot indicates that NIBAN1, SAMD7, HECTD2, and NR4A3 are significantly upregulated transcripts in the sarcopenia group (Figure 2B(Fig. 2)). The genes are primarily linked to metabolic adaptation, inflammatory response, and mitochondrial stress, indicating early transcriptional changes in sarcopenic skeletal muscle.

### Categorization of DEGs based on GO analysis

**Figure 3:**
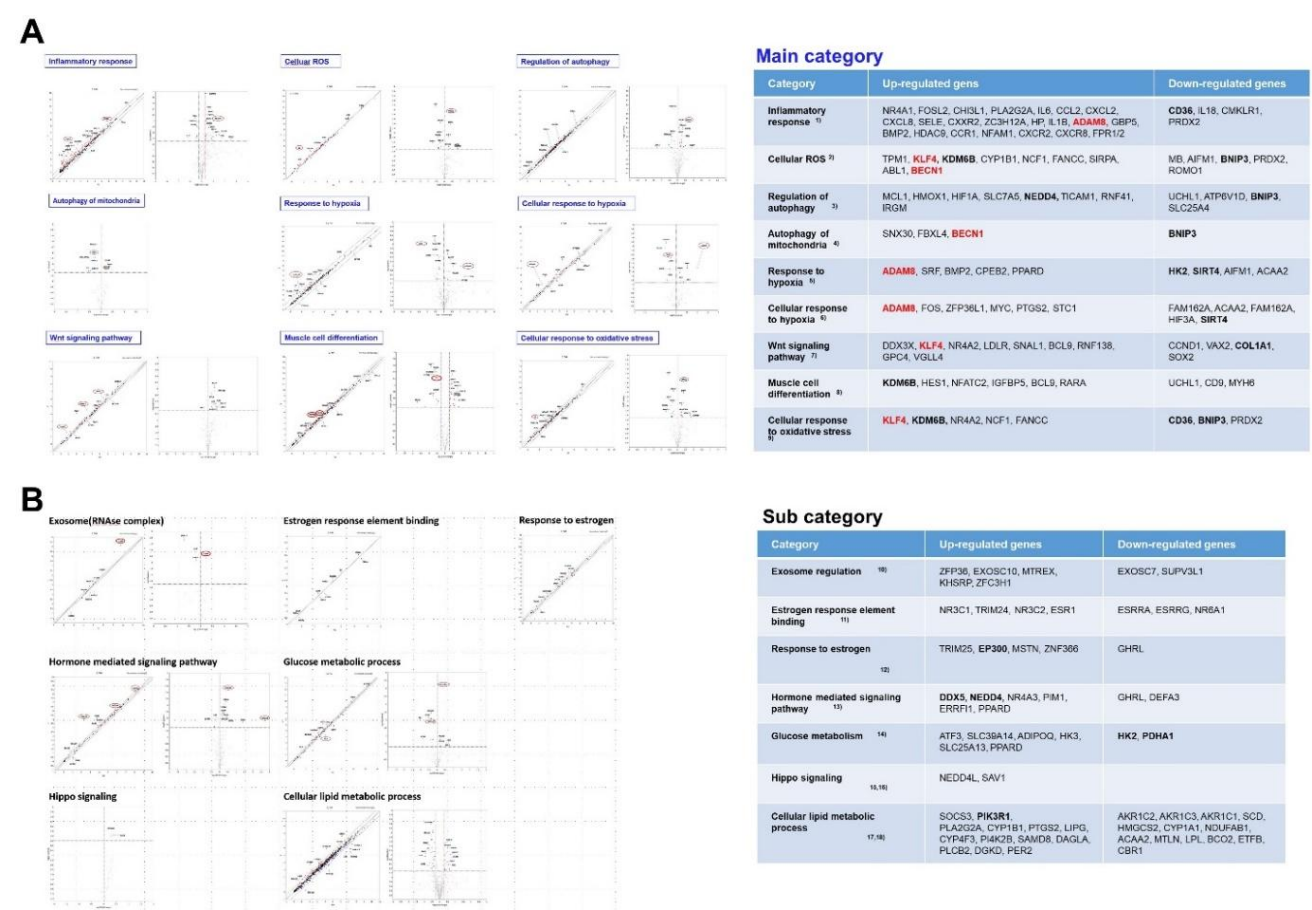
DEG analysis of the GSE226151 dataset categorized by gene ontology (GO) terms. (A) DEG were categorized according to the nine main GO terms representing the primary mechanisms of sarcopenia. The major upregulated and downregulated genes are shown in bold fonts. The most potent genes indicated the red color in the table. (B) DEG analysis graphs were categorized into seven subcategories of GO terms related to the underlying mechanisms of sarcopenia. Subcategories include exosomes (RNAse complex), estrogen response element binding, response to estrogen, hormone-mediated signaling pathway, glucose metabolic process, Hippo signaling, and cellular lipid metabolic processes. The major upregulated and downregulated genes are shown in bold fonts.

GO classification indicated that differentially expressed genes related to sarcopenia were significantly enriched in biological processes linked to inflammatory response, autophagy regulation, oxidative stress, and muscle cell differentiation (Figure 3A(Fig. 3)). The pathways are essential for sustaining skeletal muscle homeostasis. Their dysregulation signifies metabolic and immune imbalance. Seven secondary GO subcategories, including estrogen response, Hippo signaling, and lipid metabolism, were identified (Figure 3B(Fig. 3)), indicating that hormonal and energy-regulatory components contribute to the pathophysiology of sarcopenia.

### Identification of significant genes in sarcopenia: differential expression and pathway analysis 

**Figure 4:**
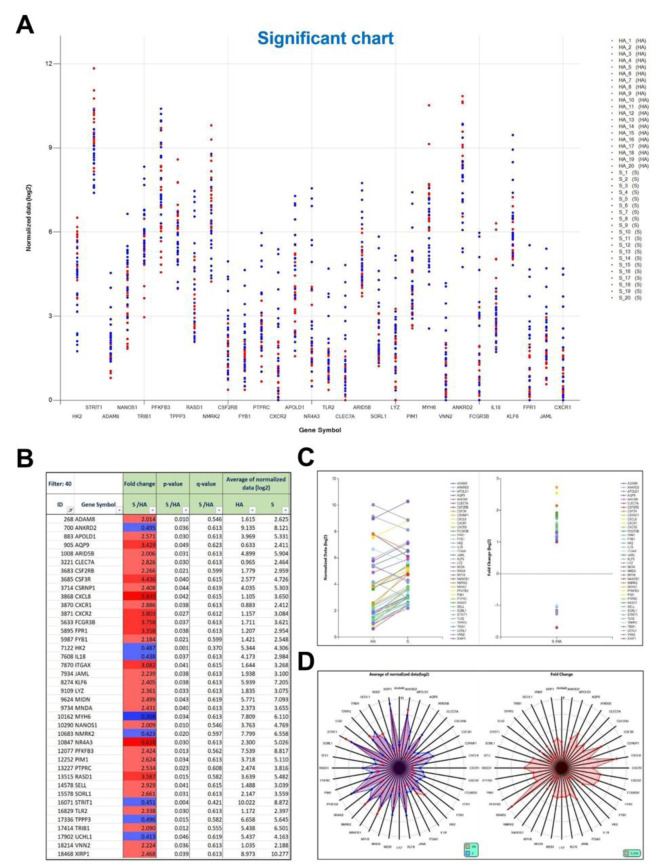
Significance plot analysis of fold change data in GSE226151 using ExDEGA and graphs and radar plots of key differentially expressed genes (DEGs) in the sarcopenia dataset. (A) The significance plot presents the fold-change analysis of the 30 key sarcopenia-regulating genes in the GSE226151 series. The x-axis represents the genes analyzed (*HK2, STRIT1, ADAM8, NANOS1, TRIB1, PFKB3, TPPP3, RASD1, NMRK2, CSF2RB, FYB1, PTPRC, CXCR2, APOLD1, NR4A3, TLR2, CLEC7A, ARID5B, SORL1, LYZ, PIM1, MYH6, VNN2, ANKRD2, FCGR3B, IL18, KLF6, FPR1, JAML, and CXCR1*). Blue dots represent genes with higher expression in the sarcopenia (S) group, such as *PFKFB3, STRIT1*, and *KLF6*. Red dots indicate lower gene expression in the healthy aged (HA) group, with the reverse pattern for increased values due to default settings in the ExDEGA program. (B) ExDEGA analysis results showing the top 40 (DEG) based on fold-change values in GSE226151: Upregulated genes are shaded in red, and downregulated genes are shaded in blue. Among the 40 genes, 32 were upregulated and only eight genes were downregulated, indicating a higher proportion of upregulated genes in the sarcopenia group. (C) Selected gene plot for the S vs. HA samples in GSE226151, showing expression patterns of sarcopenia-associated genes in the ExDEGA program: Genes with increased expression patterns include *ADAM8, AQP9, ARID5B, CLEC7A, CXCL8, CXCR1, CXCR2, IL8, KLF6, LYZ, MYH6, NANOS1, NR4A3, RASD1, SELL, TLR2, TPPP3, TRIB1, UCHL1, VNN2*, and *XIRP1*. The downregulated genes included *ANKRD2, APOLD1, CSF3R, CSF2RB, ITGAX*, and *HK2*. (D) Radar plot of the major DEGs in GSE226151: The left-side chart shows a circular plot based on the mean of normalized log values, whereas the right-side chart displays only the upregulated genes in a radar plot based on fold-change values. Genes with notably high expression levels, *NR4A3, CXCL8, CSF3R, FCGR3B, CXCR2, FPR1, ITGAX, AQP9, XIRP1, RASD1, SELL*, and *SORL1*, are highlighted.

To identify significant genes, the HA and S sets were compared. Among the 19,350 genes analyzed using the ExDEGA program, approximately 40 exhibited significant changes (Figure 4A(Fig. 4)). A significance plot analysis was used to identify 30 genes. In the S set, *PFKFB3, STRIT1,* and *KLF6* showed notably high expression. Genes with higher values in the S set (represented by blue dots in Figure 4A(Fig. 4)) were identified as potential upregulated genes in sarcopenia, including *ADAM8, NANOS1, TRIB1, PFKB3, RASD1, CSF2RB, FYB1, PTPRC, APOLD1, NR4A3, TLR2, CLEC7A, ARID5B, LYZ, PIM1, VNN2, FCGR3B, KLF6, FPR1, JAML*, and *CXCR1*. These genes were then subjected to KEGG and STRING analyses to identify their associated signaling pathways (Figure 4A(Fig. 4)). The table shows the ExDEGA analysis results displaying the top 40 DEGs based on gene expression change values in GSE226151 (Figure 4B(Fig. 4)). Upregulated genes are shown in red, and downregulated genes are shown in blue. Among the 40 significantly different genes, 32 were upregulated and only 8 were downregulated, indicating a higher proportion of upregulated genes in the sarcopenia group. The selective gene plot results for S vs. HA samples from GSE226151, showing the expression patterns of sarcopenia-related genes in the ExDEGA program (Figure 4C(Fig. 4)). Genes exhibiting increased expression patterns include ADAM8, AQP9, ARID5B, CLEC7A, CXCL8, CXCR1, CXCR2, IL8, KLF6, LYZ, MYH6, NANOS1, NR4A3, RASD1, SELL, TLR2, TPPP3, TRIB1, UCHL1, VNN2, and XIRP1. Downregulated genes include ANKRD2, APOLD1, CSF3R, CSF2RB, ITGAX, and HK2. The relatively greater number of upregulated genes reflects the complex and diverse mechanisms underlying the pathogenesis of muscular dystrophy. Radar plots of major differentially expressed genes in GSE226151 are shown in Figure 4D(Fig. 4). The left chart displays a circular plot based on the mean of normalized log values, while the right chart shows only upregulated genes in a radar plot based on fold change values. Genes with significantly elevated expression levels, including NR4A3, CXCL8, CSF3R, FCGR3B, CXCR2, FPR1, ITGAX, AQP9, XIRP1, RASD1, SELL, and SORL1, are highlighted.

### Changes in the expression of key DEGs

**Figure 5:**
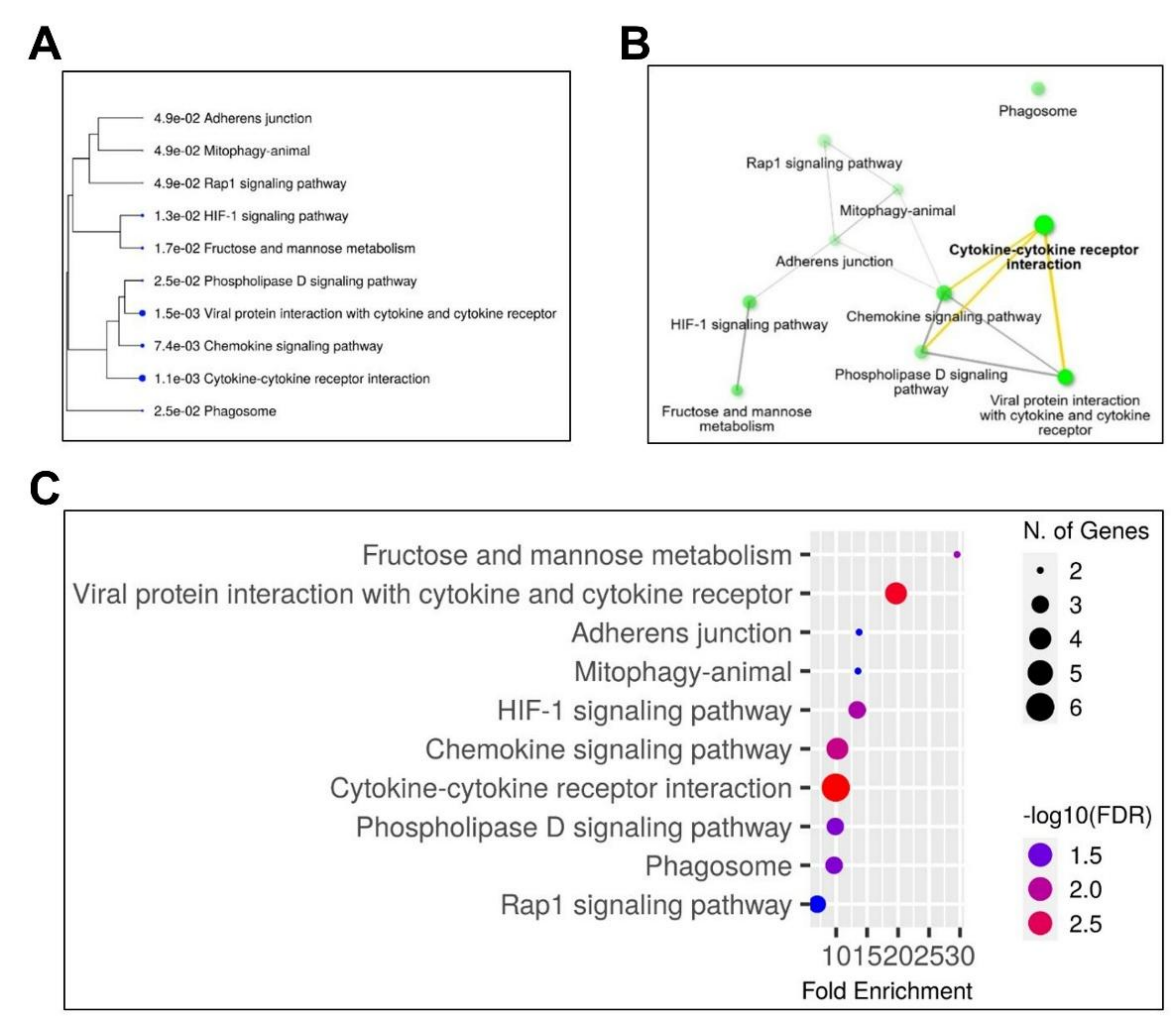
KEGG, Network, and Bar Plot Analysis of Sarcopenia Regulatory Candidate Genes (A) Tree plot from KEGG analysis of signaling pathways associated with candidate genes. This plot illustrates the KEGG analysis results for the 40 major genes implicated in sarcopenia, including those identified as upregulated in the selected dataset. The cytokine-cytokine receptor interaction pathway was identified as the primary signaling pathway associated with these candidate genes. (B) Network analysis results for the candidate genes. A network connectivity graph was generated using the KEGG analysis server (ShinyGO 0.8). The size of the green circular nodes represents the relevance of each signaling pathway. The cytokine-cytokine receptor interaction pathway has emerged as the central pathway in regulating sarcopenia, with the chemokine signaling pathway identified as the second most significant. (C) Bar plot of candidate genes: This bar plot visualizes the key signaling pathways with color-coded and sized nodes based on their significance. While the viral PPI pathway had the highest false discovery rate (FDR), the cytokine-cytokine receptor interaction pathway was considered the most significant based on its larger node size, indicating its strong relevance in sarcopenia regulation.

Of the 19,350 analyzed transcripts, 40 DEGs demonstrated significant expression differences between the S and HA groups (Figure 4A-B(Fig. 4)). Genes that exhibited significant upregulation included ADAM8, PFKFB3, RASD1, and NR4A3, while MYH6 and HK2 showed notable downregulation. The genes were analyzed using KEGG and STRING to determine the pathways and interactions that elucidate their regulatory functions in sarcopenia. KEGG enrichment analysis indicated significant participation in cytokine-cytokine receptor interaction, phospholipase D, and chemokine signaling pathways, highlighting immune-mediated and metabolic dysregulation (Figure 5A-B(Fig. 5)).

### KEGG, Network, and Gene Enrichment Analyses

KEGG pathway enrichment analysis (Figure 5A-C(Fig. 5)) identified cytokine-cytokine receptor interaction as the most significant pathway (FDR < 0.05), encompassing genes including CXCL8, CXCR2, and TLR2. Other pathways involved chemokine signaling and phospholipase D signaling, indicating immune cell recruitment and changes in lipid metabolism. The visualization network showed that these pathways contain overlapping genes that mediate inflammatory and oxidative stress responses, which are critical processes associated with muscle degeneration in sarcopenia.

### STRING Analysis of Candidate Genes

**Figure 6:**
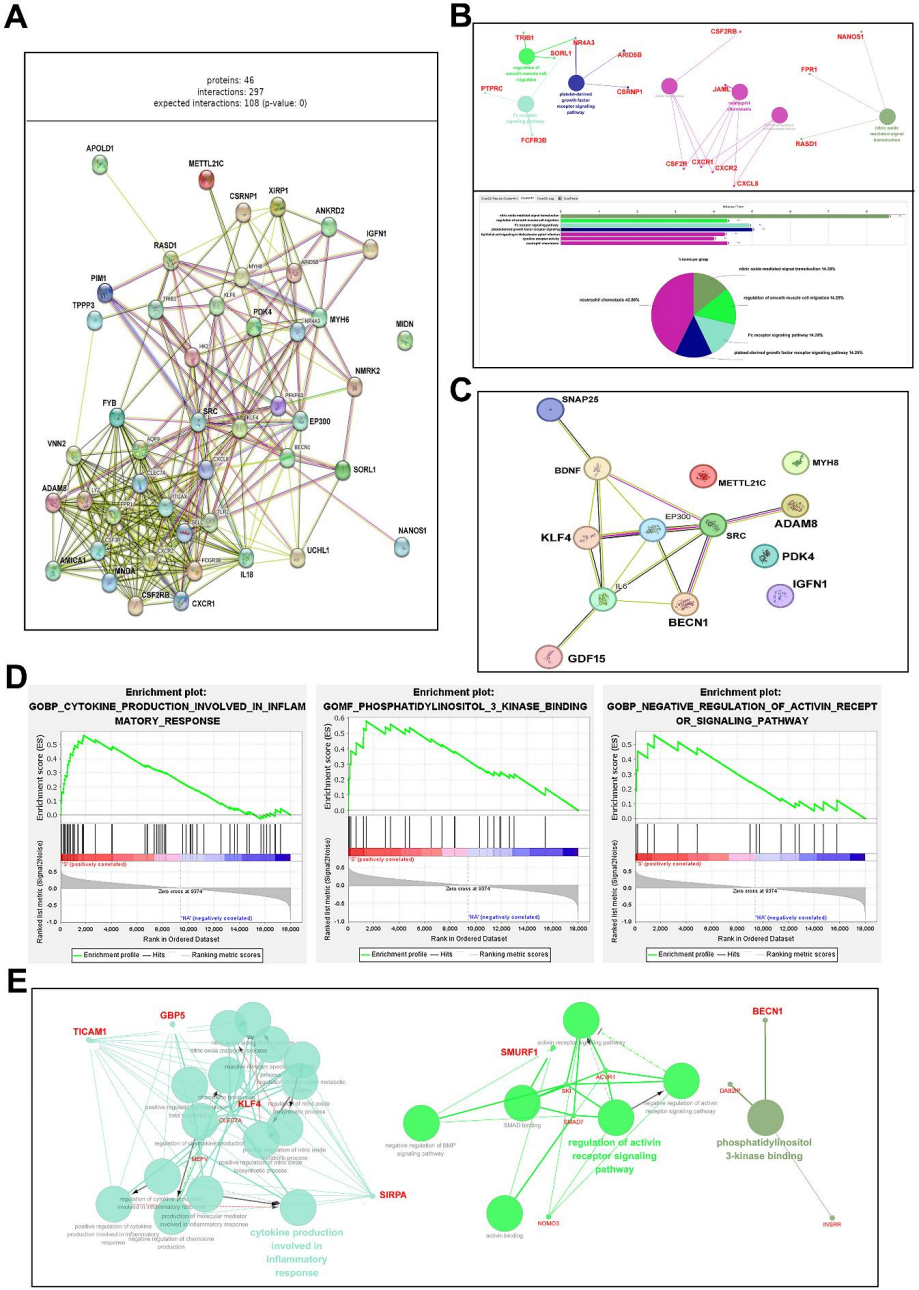
Interaction and pathway analyses of candidate sarcopenia genes and GSEA and key PPI analysis in Sarcopenia GSE226151. (A) STRING analysis results of 46 candidate genes: This analysis included 40 candidate genes identified from the GSE226151 dataset, along with six additional genes (*MYH8, PDK4, METTL21C, IGFN1, SRC,* and *EP300*) to examine protein-protein interaction (PPI) regulation. (B) PPI results of selected candidate genes: Out of 19,350 genes, 46 were initially extracted for PPI analysis, and 13 candidate genes were further selected based on PCA analysis. The selected genes included *SNAP25, BNDF, KLF4, IL6, BECN1, EP300, SRC, ADAM8, GDF15, PDK4, IGFN1, MYH8*, and *METTL21C*. *KLF4, EP300, SRC*, and *ADAM8* are identified as central nodes of interaction. (C) Intracellular pathway analysis and Cytoscape analysis of candidate genes: In the neutrophil chemotaxis pathway, *JAML* is the closest gene to other candidates, while *CSF2RB* is the most distantly related. The platelet-derived growth factor receptor signaling pathways include *NR4A3*, *ARID5B*, and *CSRNP1*. (D) Three primary signaling pathways identified through GSEA and their respective top candidate genes based on enrichment scores. The main biological pathways involved in sarcopenia regulation are (i) cytokine production involved in the inflammatory response, (ii) phosphatidylinositol 3-kinase binding, and (iii) negative regulation of the activin receptor signaling pathway. The top five candidate genes with the highest enrichment scores in each pathway were selected as the top five genes with the highest weights based on their enrichment scores within each of the three pathways: (i) GBP5, SIRPA, CLEC7A, TICAM1, and MEFV; (ii) INSRR, PI3KR1, IRD1, DAB2IP, and BECN1; and (iii) SMAD7, ACVR1, *SMURF1*, *SKI*, and *NOMO3*. (E) Cytoscape analysis of key pathways for GSEA candidate genes. PPI signaling relationships and biological functions were inferred for each of the 15 candidate genes ranked high in the GSEA enrichment score (*GBP5, SIRPA, CLEC7A, TICAM1, MEFV, INSRR, PI3KR1, IRD1, DAB2IP, BECN1, SMAD7, ACVR1, SMURF1, SKI*, and *NOMO3*). Using the GO app in Cytoscape, a relational graph of gene associations was created, with nodes of varying sizes and colors representing gene significance and function. In the cytokine production pathway related to the inflammatory response, *KLF4, CLEC7A*, and *MEFV* appeared as central genes, whereas *TICAM1* and *SIRPA* were less prominent. Black arrows indicate the forward activation of the signaling pathway.

Analysis of 46 candidate genes using STRING identified several protein-protein interaction clusters, with ADAM8, KLF4, BECN1, and GBP5 demonstrating the highest connectivity (degree ≥ 10) (Figure 6A-C(Fig. 6)). ClueGO analysis revealed significant functional modules associated with neutrophil chemotaxis, autophagy regulation, and smooth muscle cell migration, which are critical for muscle maintenance and regeneration.

### GSEA of clinical sarcopenia samples

GSEA validated the enrichment of pathways associated with sarcopenia, aligning with findings from DEG and KEGG analyses. Key pathways included cytokine production related to the inflammatory response, PI3K-Akt signaling, and the negative regulation of activin receptor signaling (Figure 6D(Fig. 6)). GBP5 and ADAM8 participated in cytokine production, and BECN1 was associated with autophagy and PI3K binding. At the same time, SMAD7 regulated activin receptors. The genes identified collectively signify the convergence of inflammation, autophagy, and regenerative deficits in sarcopenic muscle.

Cytoscape network visualization (Figure 6E(Fig. 6)) revealed significant regulatory interactions among CLEC7A, KLF4, MEFV, and BECN1. CLEC7A functioned as a pivotal component in cytokine-related signaling, whereas BECN1 connected autophagy and PI3K-Akt pathways. The networks identified ADAM8, BECN1, KLF4, and GBP5 as significantly interconnected genes associated with immune signaling, oxidative stress, and muscle regeneration.

Collectively, the primary signaling pathways associated with sarcopenia-related gene expression were identified as the inflammatory response, cellular ROS response, and skeletal muscle tissue regeneration. Within the inflammatory response pathway, the upregulated genes included *ADAM8*, *CLEC7A*, *CXCL8*, *CXCR2*, *LYZ*, and *TLR2*. For the cellular ROS response, the newly identified candidate genes encompassed *GBP5, ADAM8, HDAC9, NFAM1*, and *FPR1*. In the skeletal muscle regeneration pathway, *XIRP1* was highlighted as a significant gene. Additionally, genes involved in skeletal muscle contraction included upregulated regulators, such as *ACTN3*, *TRIM63*, and *CFLAR*, whereas *TNNC1*, *DMPK*, and *MTH7* were identified as negative regulators of muscle contraction.

## Discussion

Sarcopenia remains a multifactorial disorder marked by intricate interactions among metabolic, inflammatory, and degenerative pathways. Its onset and progression are influenced by intrinsic factors such as age, sex, physical activity, diet, and comorbidities, as well as extrinsic factors such as environment and lifestyle (Yuan et al., 2023[30]). The diversity complicates the distinction between primary sarcopenia, which is associated with aging, and secondary forms that arise from chronic diseases or malnutrition (Wiedmer et al., 2021[26]). Primary sarcopenia is mainly associated with aging and is often exacerbated by disease or lifestyle factors, while secondary sarcopenia results from non-age-related muscle loss. While numerous potential biomarkers have been identified through serum and tissue analyses, the majority of mechanistic insights have originated from animal models, and the availability of human molecular data, especially within Asian populations, remains limited. Histological evaluation is the conclusive diagnostic method; however, it is limited by the invasive nature of muscle biopsies (Wilson et al., 2018[27]). In this context, the present study enhances the understanding of sarcopenia through an integrative transcriptomic analysis aimed at identifying key regulatory genes from the Asian-origin dataset GSE226151. An analysis of approximately 19,000 genes from vastus lateralis muscle samples identified 40 DEGs associated with inflammatory, oxidative, and autophagic processes. 

Figure 2(Fig. 2) shows that DEG profiling identified 40 genes that were significantly altered between the S and HA groups, indicating substantial transcriptional remodeling. GO and KEGG enrichment analyses (Figure 3-5(Fig. 3)(Fig. 4))(Fig. 5) indicated that these genes are predominantly associated with pathways related to inflammation, oxidative stress, and autophagy. The enhancement of cytokine-cytokine receptor interactions and chemokine signaling pathways underscores the role of chronic low-grade inflammation, known as “inflammaging,” in muscle atrophy (Sakuma et al., 2017[22]). Pro-inflammatory cytokines, including IL-6, TNF-α, and IL-1β, keep the NF-κB pathway active, leading to increased proteolysis and reduced anabolic signaling. This, in turn, breaks down muscle. The upregulated inflammatory mediators-CXCL8, CXCR2, CLEC7A, and ADAM8-identified in this dataset support the notion that prolonged immune activation disrupts muscle homeostasis, possibly hastening mitochondrial failure and hindering regeneration.

GSEA provided detailed insights into gene enrichment within these pathways. The top-ranked genes included *GBP5, SIRPA, CLEC7A, TICAM1*, and *MEFV* in cytokine production within the inflammatory response*; INSRR, PI3KR1, IRD1, DAB2IP*, and *BECN1* in PI3K binding; and *SMAD7*, *ACVR1*, *SMURF1*, *SKI*, and *NOMO3* in negative regulation of the activin receptor signaling pathway. While discrepancies emerged between the DEG and GSEA findings, these differences could be attributed to limitations in analytical methodologies rather than to inherent flaws.

STRING analysis further elucidated the interactions and associated pathways among the candidate genes. *CLEC7A* has been identified as central to cytokine production in the inflammatory response pathway, with strong links to *MEFV* and *KLF4*. In the activin receptor signaling pathway, *ACVR1,* *SKI*, and *SMAD7* demonstrated close interactions, whereas *NOMO3* was relatively distant. Within the PI3K binding pathway, *DAB2IP* was prominently positioned. Overall, the findings highlight critical pathways associated with sarcopenia, including the inflammatory response, activin receptor signaling, and PI3K signaling. Inflammatory pathways are characterized by elevated levels of pro-inflammatory cytokines, such as IL-1β, IL-6, TNF-α, and CRP, and decreased levels of anti-inflammatory cytokines, such as IL10. Genes upregulated in sarcopenia-related inflammatory responses included *ADAM8, CLEC7A, CXCL8, CXCR2, LYZ*, and *TLR2*. Overall, the analysis of PPI signaling relationships and biological functions inferred for the 15 candidate genes with high GSEA enrichment scores identified *ADAM8*, *BECN1*, *KLF4*, and *GBP5* as key regulatory candidates, with increased expression linked to sarcopenia.

Among the hub genes, ADAM8 is distinguished as a multifunctional membrane metalloproteinase that modulates cytokine shedding, extracellular matrix remodeling, and immune cell migration. ADAM8 was previously associated with inflammatory conditions, including asthma (King et al., 2004[13]), and is a potential therapeutic target in pancreatic cancer (Schlomann et al., 2015[23]). The increase in sarcopenic muscle indicates ongoing immune activity and matrix change, potentially undermining tissue stability. ADAM8 may serve as a molecular link between immune cell infiltration and structural deterioration, accounting for the persistent microinflammatory phenotype observed in aged muscle. BECN1 plays a pivotal role in autophagy and in regulating the PI3K pathway (Kang et al., 2011[10]). Autophagy plays a critical role in the recycling of damaged proteins and mitochondria; however, its decline with age results in the accumulation of dysfunctional organelles and increased oxidative stress. The observed enrichment of the PI3K-Akt signaling pathway in this study suggests that impaired autophagy plays a significant role in the pathogenesis of sarcopenia. Prior studies have linked impaired PI3K/Akt/mTOR signaling with anabolic resistance and reduced protein synthesis in elderly individuals (McKnight et al., 2013[16]). Animal studies have demonstrated a close relationship between the PI3K-Akt pathway and muscle synthesis (Abdelrahman et al., 2023[1]). Most human studies on sarcopenia suggest that a diminished synthetic response, commonly referred to as anabolic resistance, is a key factor in the loss of muscle mass (Wall et al., 2015[25]). BECN1 has also been linked to infectious diseases and neurodegenerative disorders such as Alzheimer's disease. The robust connectivity of BECN1 within our network underlines its function as a molecular switch that regulates cell survival and apoptosis. Dysregulated Beclin-1 activity may therefore expedite the shift from adaptive autophagy to cell death, leading to muscle wasting.

KLF4, a zinc-finger transcription factor involved in cell differentiation and the stress response, has been identified as a hub gene. The observed upregulation of KLF4 in sarcopenic tissue suggests an adaptive yet maladaptive transcriptional reprogramming that promotes fibro-adipogenic differentiation at the expense of myogenic repair. The connection to transcriptional coactivators like EP300 and CREBBP (Evans et al., 2007[6]) suggests a possible epigenetic regulation of inflammatory gene expression in sarcopenia, a hypothesis that requires experimental verification. 

GBP5, an interferon-inducible guanylate-binding protein, was identified as a significant hub gene through STRING and GSEA analysis (Figure 6E(Fig. 6)). GBP5 activates the NLRP3 inflammasome and regulates macrophage polarization, thereby promoting the secretion of IL-1β and IL-18 in chronic inflammation (Lu et al., 2025[15]). The upregulation observed in sarcopenic samples suggests a persistent sterile inflammatory response. Increased GBP5 expression may support macrophage-mediated cytokine production, hindering tissue repair and biasing macrophage populations towards an M1-like phenotype. This finding corresponds with the inflammatory dominance noted in aged muscle and may elucidate the impaired resolution of inflammation during regeneration.

Integrative analysis of DEG, KEGG, STRING, and GSEA data shows that chronic inflammation, defective autophagy, and impaired regenerative signaling are linked. PI3K-Akt signaling pathways and cytokine-cytokine receptor contact suggest a self-amplifying feedback loop between inflammatory stress and autophagy failure. Pro-inflammatory cytokines restrict autophagic flux, whereas cellular damage sustains cytokine production, creating a catabolic dominance loop. Figure 6C-D(Fig. 6) shows that ADAM8 (inflammation) and BECN1 (autophagy regulation) are interdependent, whereas KLF4 and GBP5 integrate transcriptional and immunological components of the sarcopenic phenotype. 

String analysis showed differential grouping between S and HA samples, demonstrating transcriptional segregation and validating the gene panel's diagnostic potential. In Asian populations, ADAM8, BECN1, KLF4, and GBP5 may be predictive biomarkers for early sarcopenia identification. This is important because ethnicity and genetics affect muscle function and disease susceptibility.

While transcriptomic studies can reveal molecular signatures linked to disease states, the constrained sample size limits statistical power and external validity, and dependence solely on transcriptomic data hinders the establishment of causal relationships between gene expression changes and pathophysiology. It is crucial to obtain experimental confirmation through both in vitro and in vivo models to clarify the precise mechanistic activities of these hub genes. The integration of multi-omics data-including proteomic, epigenomic, and metabolomic data-has the potential to enhance our understanding of the pathophysiology of sarcopenia.

## Conclusion

This study conducts a thorough transcriptomic analysis, identifying ADAM8, BECN1, KLF4, and GBP5 as critical regulatory genes that manage the inflammatory, autophagic, and regenerative networks associated with sarcopenia. The connections illustrated in Figures 5-6(Fig. 5)(Fig. 6) indicate a cohesive molecular mechanism in which inflammaging, mitochondrial dysfunction, and transcriptional reprogramming collectively contribute to muscle atrophy. The findings establish a foundation for biomarker-guided diagnostics and treatment approaches that address sarcopenia and enhance the quality of life for older adults. ADAM8 and GBP5 are linked to ongoing immune activation, BECN1 is associated with impaired mitochondrial quality control, and KLF4 is connected to modified transcriptional regulation of myogenesis.

This integrative transcriptomic approach, despite limitations in sample size and absence of experimental validation, provides a biologically consistent framework that improves the understanding of sarcopenia pathophysiology. The findings identify these four genes as potential biomarkers and therapeutic targets, providing opportunities for early diagnosis and precision interventions. The study addresses ethnic disparities in sarcopenia research and lays the foundation for strategies to mitigate muscle loss and promote healthy aging.

## Notes

Jae Gyu Kim, Ashish Ranjan Sharma, and Yeon-Hee Lee contributed equally as first author.

## Declaration

### Acknowledgements 

This study was supported by Hallym University Research Fund, the Basic Science Research Program through the National Research Foundation of Korea (NRF) funded by the Ministry of Education (NRF-2020R1C1C1008694, RS-2023-00272748 & RS-2025-00513909). We further acknowledge the German Federal Ministry of Research, Technology, and Space (BMFTR) for funding CarBoMD (Grant ID 13XP5206).

### Conflicts of interest

The authors have declared no conflict of interest regarding the publication of this paper.

### Author contributions

JGK and SSL conceived and designed the manuscript. JGK, ARS, and YHL drafted the manuscript. JGK, ARS, YHL, and MJK contributed to the investigation, data curation, and visualization. CC and HJ reviewed the manuscript. SSL contributed to supervision of review writing, funding acquisition, and project administration. All authors have approved the final version of the manuscript.

### Using Artificial Intelligence (AI) 

The intelligibility and clarity of this work were enhanced through the utilization of artificial intelligence (AI)-powered language models. The authors are solely responsible for the content of the manuscript, as they reviewed and edited all AI-assisted text.

### Data availability

The datasets analyzed during the current study are available in the GEO repository (https://www.ncbi.nlm.nih.gov/geo/.), accession number GSE226151.

## Supplementary Material

Supplementary data

## Figures and Tables

**Table 1:**
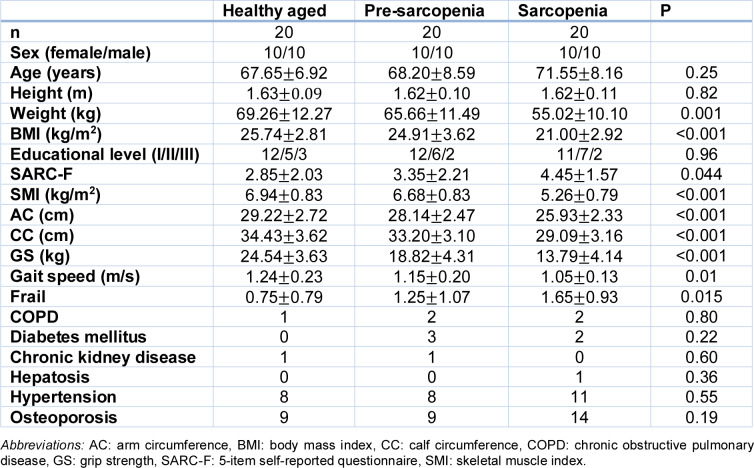
Demographics and baseline characteristics of participants, as listed in the GEO database (GSE226151 series; GEO-NCBI, nih.gov), retrieved on February 22, 2024.

**Figure 1:**
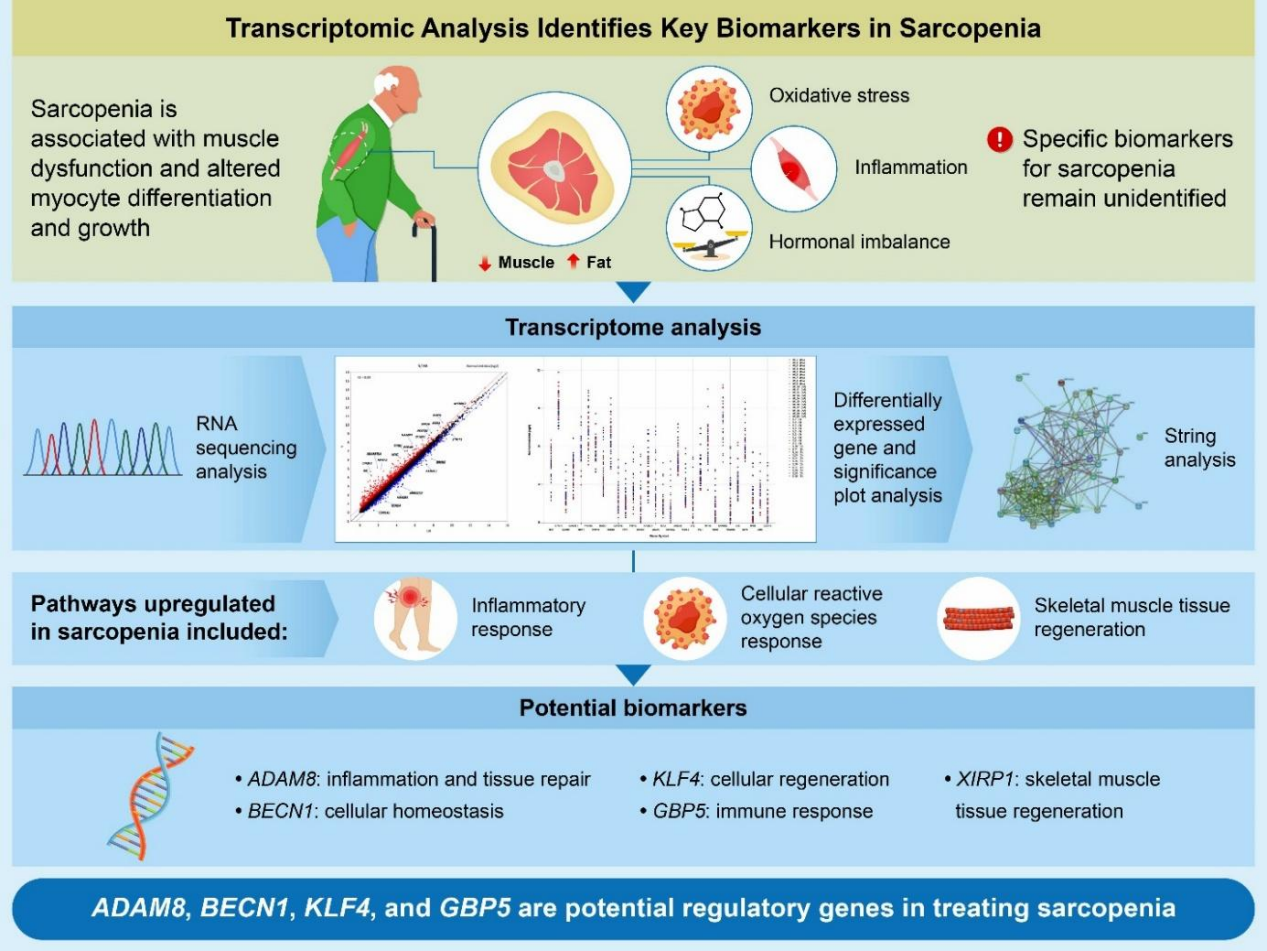
Graphical abstract. Schematic diagram demonstrating the identification of key hub genes linked to sarcopenia from Asian transcriptome data using bioinformatic analysis.
